# Adenosine induced ventricular fibrillation in a structurally normal heart: a case report

**DOI:** 10.1186/s13256-016-1177-z

**Published:** 2017-01-22

**Authors:** Christopher A. Rajkumar, Norman Qureshi, Fu Siong Ng, Vasileios F. Panoulas, Phang Boon Lim

**Affiliations:** 1 Imperial College Healthcare NHS Trust, Hammersmith Hospital, London, W12 0HS UK; 2 Imperial College London, Hammersmith Hospital Campus, London, W12 0NN UK

**Keywords:** Adenosine, Atrial fibrillation, Ventricular fibrillation

## Abstract

**Background:**

Adenosine is the first-line pharmacotherapy for termination of supraventricular tachycardia through its action on the atrioventricular node. However, pro-arrhythmic effects of adenosine are also recognised, most notably in the presence of pre-excited atrial fibrillation. In this case report, we describe the induction of ventricular fibrillation in a patient with no demonstrable accessory pathway, nor any other structural heart disease. This rare, idiosyncratic reaction has never previously been reported and is of relevance given the widespread and routine use of adenosine in clinical practice.

**Case presentation:**

A 26-year-old woman of Cypriot origin presented to our emergency department with a sudden onset of palpitations and chest discomfort. She was healthy, with no previous medical history and no regular medications. An electrocardiogram demonstrated a narrow complex tachycardia with a rate of 194 beats per minute. Following failure of vagal maneuvers to terminate the tachycardia, the assessing physician administered a single intravenous dose of 6 mg adenosine. Our patient instantaneously developed coarse ventricular fibrillation and circulatory collapse. Cardiopulmonary resuscitation was initiated and our patient was rapidly defibrillated to sinus rhythm with a single 150 J direct current shock. A 900-mg loading dose of intravenous amiodarone was commenced and our patient was managed in the cardiac high dependency unit. No further arrhythmias were identified on continuous cardiac monitoring.

On review, her presenting electrocardiogram had demonstrated rapidly conducted atrial fibrillation with no evidence of ventricular pre-excitation. Concordantly, her resting electrocardiogram was not suggestive of any accessory pathway. This was conclusively excluded on invasive electrophysiology study, with negative programmed ventricular stimulation up to three extrastimuli. Extensive laboratory investigations were unremarkable and failed to identify an underlying cause for her episode of atrial fibrillation. Furthermore, cardiac magnetic resonance imaging demonstrated a structurally normal heart, with no edema, fibrosis or infarction as well as normal coronary artery anatomy.

**Conclusions:**

Adenosine remains a safe and highly efficacious therapy for supraventricular tachycardia. However, this unusual case demonstrates the ability of adenosine to induce circulatory collapse and reminds the clinician that prompt access to resuscitation, defibrillation, and transcutaneous pacing equipment is mandatory with every administration of this drug.

## Background

Adenosine is an endogenous nucleoside and the first-line pharmacological agent for the diagnosis and termination of supraventricular tachycardia (SVT). The primary mechanism of action is mediated through inhibition of the conduction capabilities of the atrioventricular node. However, pro-arrhythmic effects of adenosine are increasingly recognised, suggesting a more heterogeneous pharmacodynamic profile. Recognized tachyarrhythmias following administration of adenosine include (1) induction of atrial fibrillation (AF), (2) ventricular fibrillation (VF) in pre-excited AF, (3) polymorphic ventricular tachycardia (VT) in long Q-T syndromes, (4) degeneration of VT to VF and finally, (5) non-sustained VT following termination of SVT [1]. Below, a case of ventricular fibrillation following administration of adenosine in a structurally normal heart is described. To the best of our knowledge, this is a novel finding which has not been previously reported in the literature.

## Case presentation

A 26-year-old woman of Cypriot origin presented with an acute onset of palpitations and chest tightness. She reported several weeks’ history of similar palpitations, however, this was the first occasion that the sensation had not self-terminated within minutes and had been associated with discomfort.

There was no further medical history of note, no relevant family history, and no regular medications. Our patient admitted to a moderate alcohol intake although no recent intoxication and no recreational drug use.

**Fig. 1:**
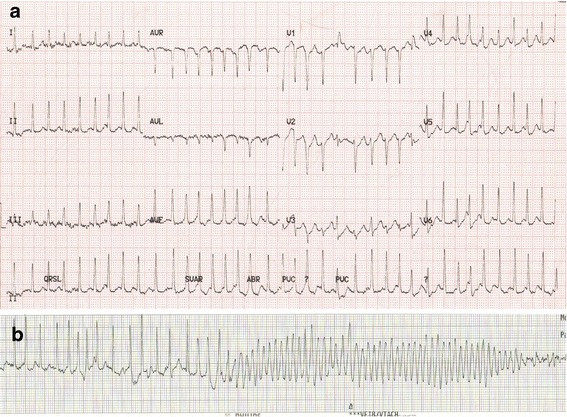
**a** Twelve-lead electrocardiogram at initial presentation. **b** Onset of ventricular fibrillation following administration of adenosine

On assessment in the Emergency Department, her electrocardiogram (ECG) demonstrated a narrow-complex tachycardia with a rate of 194 beats per minute (bpm) (Fig. 1a). Her heart rate varied between 180 and 210 bpm and her blood pressure was 187/101 mmHg on first recording. Suspecting SVT, the assessing emergency physicians proceeded with vagal maneuvers, to no clinical effect. Next, an intravenous bolus dose of 6 mg adenosine was administered. This was followed by immediate development of coarse VF (Fig. 1b) and circulatory collapse. Rapid deterioration to a fine VF ensued. She was successfully defibrillated with a single 150 J direct current shock. There was immediate return of spontaneous circulation and hemodynamic stability. A 900-mg loading dose of intravenous amiodarone was commenced and our patient was admitted to the cardiac high dependency unit. No further arrhythmias were identified.

**Fig. 2:**
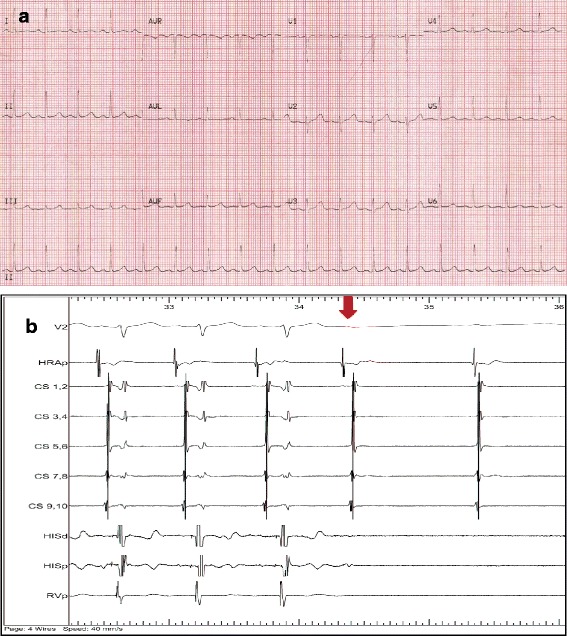
**a** Resting electrocardiogram in sinus rhythm. **b** A four-wire electrophysiology study was conducted in our patient. Twelve milligrams of adenosine was administered resulting in progressive lengthening of the PR interval (surface electrocardiogram V2), and eventual atrioventricular block (*red arrow*) with non-conducted P waves. This excluded the presence of an anterograde conducting accessory pathway. *HRA* high right atrium, *CS* coronary sinus, *HIS* His and *RV* right ventricular apex catheters

Investigations revealed normal serum electrolytes, negative serial troponin measurements, normal thyroid function, and negative blood ethanol level. A urine drug screen was negative. A chest radiograph demonstrated clear lung fields. The presenting ECG was reviewed, and was likely to be that of AF with a rapid ventricular response, but with no clear ventricular pre-excitation. Similarly, her ECG in sinus rhythm had no features of ventricular pre-excitation (Fig. 2a). The Q-Tc interval was within normal range and there were no features of Brugada syndrome. In view of the development of VF with adenosine, an electrophysiology study was performed. Following administration of adenosine, there was progressive PR lengthening and eventual atrioventricular (AV) block with non-conducted P waves, excluding a typical anterograde conducting accessory pathway (Fig. 2b). There was also a negative programmed ventricular stimulation up to three extrastimuli. On exercise stress testing, there was an appropriate heart rate and blood pressure response with no ST/T wave changes and no arrhythmias identified. Cardiac magnetic resonance imaging (MRI) confirmed normal biventricular volumes and systolic function, no myocardial edema, fibrosis or infarction, and normal origins of the right and left coronary arteries.

Our patient was discharged, without any prescribed medications, following a short inpatient stay during which no further episodes of arrhythmia were identified on continuous cardiac monitoring. At routine follow-up 3 months later, our patient remained well, with no further symptoms and no recurrence of palpitations. She was satisfied by the care she had received and was discharged from routine outpatient review.

## Discussion

The widespread use of adenosine in emergency departments is testament to a high degree of safety and efficacy in routine practice. The case described represents a rare, idiosyncratic response to adenosine with an unclear mechanism.

Ventricular ectopy and non-sustained monomorphic and polymorphic VT without hemodynamic compromise have often been reported following the administration of adenosine in structurally normal hearts [2, 3]. Furthermore, a case of adenosine-induced ventricular flutter, degenerating to VF, with concurrent administration of verapamil and digoxin has been observed as a pause-dependent phenomenon [4]. However, this is the first published case report of ventricular fibrillation with hemodynamic collapse, induced by adenosine in an otherwise healthy heart. Specifically, this case is novel in demonstrating adenosine-induced VF in the absence of bradyarrhythmia, ventricular pre-excitation or use of other anti-arrhythmic drugs.

DiMarco and colleagues [5] identified ventricular ectopy in one third of all patients following administration of adenosine, suggesting a widespread effect of increased ventricular automaticity. This finding has been supported in animal studies, whereby the pro-excitatory effects of adenosine have been isolated to the A_2_ adenosine receptor in ventricular myocytes [6]. Catecholamine release is also a precipitating factor for increased ventricular automaticity [6], and in fact adenosine has been demonstrated to activate a cyclic adenosine monophosphate (cAMP)-dependent system which serves to sensitize the myocardium to the actions of catecholamines, potentiating their effect [7]. Furthermore, adenosine administration is independently associated with a reflex increase in sympathetic tone and circulating catecholamine levels [8]. These mechanisms may, at least in part, be responsible for the development of VF with adenosine, from rapidly conducted AF in the context of an adrenergic state.

An alternative hypothesis may be found by examining the effect of adenosine on ventricular repolarization. Adenosine shortens the effective refractory period in atrial myocytes and predisposes to atrial arrhythmias such as atrial fibrillation and flutter through increased spatial dispersion of refractoriness [1]. This phenomenon, induced by adenosine, has also been demonstrated in ventricular myocardium and may predispose to ventricular arrhythmogenesis in a similar manner [9].

In this patient, a low dose of adenosine (6 mg) was able to induce a malignant ventricular arrhythmia in the setting of rapidly conducted atrial fibrillation. However, in sinus rhythm, a greater dose of adenosine (12 mg) had no such effect. This suggests that atrial fibrillation with a rapid ventricular response provides an unstable electrical substrate, likely due to irregular R-R intervals and variable ventricular refractory periods, that renders the ventricles susceptible to the pro-arrhythmic effects of adenosine, as mentioned previously.

Unfortunately, a 12-lead electrocardiogram (ECG) was not recorded in this patient at the moment of administration of adenosine. Recording a continuous 12-lead ECG during therapeutic interventions such as administration of anti-arrhythmic drugs should be encouraged, as in this case, it may have shed valuable light on the underlying mechanism for arrhythmia which could not be appreciated from a rhythm strip alone.

## Conclusions

Clinically, this unusual case serves as a reminder of the ability of adenosine to induce ventricular tachyarrhythmias associated with circulatory collapse. The well-recognised adverse effects of bronchospasm, chest pain and bradycardia, combined with the potential for ventricular arrhythmias should mandate all physicians to use adenosine only where there is prompt access to resuscitation, defibrillation, and transcutaneous pacing equipment. Nevertheless, physicians should not be deterred from using this highly efficacious agent where an appropriate assessment of the risks incurred has been borne in mind.

## Abbreviations

AF: atrial fibrillation
ECG: electrocardiogram
MRI: magnetic resonance imaging
SVT: supraventricular tachycardia
VF: ventricular fibrillation
VT: ventricular tachycardia
